# Cross‐Compartment Multimodal Analyses Reveal Differences in IL2‐STAT5 Signaling Associated With Asthma in Individuals From Diverse Backgrounds

**DOI:** 10.1002/mco2.70810

**Published:** 2026-06-11

**Authors:** Duan Ni, Ralph Nanan

**Affiliations:** ^1^ Sydney Medical School Nepean The University of Sydney Sydney New South Wales Australia; ^2^ Charles Perkins Centre The University of Sydney Sydney New South Wales Australia; ^3^ Nepean Hospital, Nepean Blue Mountains Local Health District Sydney New South Wales Australia

**Keywords:** African American, ancestral differences, asthma, IL2‐STAT5, self‐reported racial group

## Abstract

Self‐reported racial background has been associated with differences in asthma risk and severity, yet detailed insights remain limited. Non‐European American (EA)/non‐White populations are considerably underrepresented in this context. To address this gap, we performed multimodal analyses across various compartments in healthy individuals and asthma patients, interrogating potential differences related to self‐reported racial background. During asthma, self‐reported African American (AA) patients exhibited higher IL2‐STAT5 signaling, a key pathway in asthma pathophysiology, across bronchial and airway epithelium, circulating CD4^+^ T cells, whole blood, and peripheral blood mononuclear cells (PBMCs). This correlated with increased T cell activation and recruitment, processes central to asthma development. In healthy AA, compared to EA individuals, CITE‐seq, scRNA‐seq, and phospho‐CyTOF analyses revealed elevated IL2‐STAT5 signaling in PBMCs and lung‐derived immune cells, suggesting potentially heightened predispositions toward asthma. Together, these findings provide initial insights suggesting self‐reported racial background‐related differences in immune pathways relevant to asthma, highlighting the functional implication of IL2‐STAT5 signaling. If validated in larger and more diverse cohorts, these insights may inform risk stratification strategies and targeted therapeutic approaches. Our results highlight the urgent need for broader representation of diverse background populations in asthma research to advance more equitable and biologically informed precision medicine.

## Graphical abstract

Cross‐compartment multimodal analyses reveal that under both healthy and asthmatic conditions, self‐reported African American (AA) individuals are linked to upregulation of IL2‐STAT5 signaling compared with self‐reported European American (EA) individuals, which is linked to enhanced T cell activation and recruitment during asthma. These findings highlight the necessity of greater diversity in asthma research to advance equitable healthcare solutions.

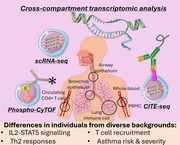

## 1 Introduction

The influence of ethnicity/race/ancestry is increasingly recognized as an important, yet complex and sometimes controversial consideration in biomedicine. These nomenclatures and definitions continue to evolve and change. At present, *race* and *ethnicity* are generally understood as sociocultural constructs rather than biological categories, while *ancestry* may carry greater biological relevance, as it refers to a person's country or region of origin, or their lineage of descent [1, 2]. Previous studies have suggested that self‐reported racial groupings can, to some extent, align with underlying genetic profiles [3]. However, more recent work has challenged such relationship, highlighting the need for careful interpretation [4]. Together, clear distinctions among individuals’ self‐reported racial groupings, ancestral backgrounds and genetic backgrounds are important when examining population‐level differences in health and disease and they should be considered in their respective specific contexts. Historically, most biomedical studies have documented self‐reported *race* or *ethnicity*, while information on *ancestry* has been collected less consistently. Although the use and interpretation of these classifications remain debated, investigations in this area are increasingly recognized as important and valuable. Such research has the potential to help to identify and address health disparities and inequities [1, 2, 5, 6, 7]. As a result, these factors are of scientific interest and clinical significance, especially in pursuit of personalized medicine and improving population health outcomes.

Here, we adopt the term *self‐reported racial group*, which is commonly used in prior biomedical research, to examine the potentially relevant differences in disease pathophysiology and treatment responses. Accumulating evidence from oncology is exemplary for the importance of this approach. For example, studies have reported differences between self‐reported Black versus White breast cancer patients [8] in tumor‐infiltrating lymphocytes, variations in immune checkpoint PD‐L1 signaling between self‐reported African American (AA) versus Hispanic breast cancer patients [9], and distinct immune‐related signaling pathways in self‐reported African and European patients with prostate cancer [10]. Consistent with these observations, a recent study documented profound difference in patient responses to cancer immunotherapy [11] associated with self‐reported racial groups. Differences associated with self‐reported racial groups have also been described in other immune‐related diseases, including autoimmune diseases. For example, self‐reported Black women have a higher risk of developing systemic lupus erythematosus and often exhibit more severe disease manifestations relative to self‐reported White women. This pattern might at least in part be explained by differences in their immune phenotypes [12]. In multiple sclerosis, higher prevalence rates have been reported in self‐reported White and Black racial groups compared to self‐reported Hispanic and Asian racial groups [13]. Lately, in the context of atopic diseases, we identified immune signaling differences in atopic dermatitis and in response to dupilumab treatment across self‐reported racial groups [14]. These findings all support the relevance of considering self‐reported racial background in biomedical studies, particularly when investigating immune‐related diseases.

Asthma is another major atopic disease. Its patterns of risk, prevalence, and severity also appear to vary across self‐reported racial groups. Some studies suggest that individuals of self‐reported AA background are at higher risk of asthma and tend to experience more severe disease manifestations [15, 16, 17, 18, 19, 20, 21]. In contrast, a survey based on the Medical Research Council DASH (Determinants of Adolescent Social well‐being and Health) study found a lower prevalence of asthma among self‐reported Black African boys and girls [22]. These inconsistencies highlight the lack of consensus in the field. One of the contributing factors to this conundrum may be that majority of the existing asthma research has focused predominantly on self‐reported Caucasian/White/European American (EA) racial groups, with more limited representation of individuals from more diverse backgrounds. Additionally, studies examining differences across self‐reported racial groups have largely been epidemiological and observational, while the underlying detailed molecular insights remain poorly understood. In this study, we therefore aim to elucidate the potential detailed differences in asthma‐related signaling pathways among individuals of different self‐reported racial groups.

We postulated that pre‐existing multimodal datasets from individuals of diverse backgrounds, across multiple organ compartments, could help identify biological differences across self‐reported racial groups. Such differences may potentially be associated with their varying risk profiles for asthma development and exacerbation, as previously observed [15, 16, 17, 18, 19, 20, 21, 22]. To test this, we carried out a comprehensive cross‐compartment, multimodal analyses, comparing individuals from different self‐reported racial groups under both healthy and asthma conditions based on publicly available data. An extensively survey of publicly available multimodal datasets found a clear imbalance of representation: self‐reported EA individuals represented the majority of the participants involved, followed by self‐reported AA individuals, while other self‐reported racial groups were substantially underrepresented. Due to this underrepresentation of other self‐reported racial groups, it was statistically not possible to include them in our analyses. Cross‐modal analyses revealed that under both asthma and healthy conditions, self‐reported AA individuals consistently showed enhanced IL2‐STAT5 signaling, a pathway known to play a central role in asthma pathophysiology, across various organ systems. These findings suggest a potential biological predisposition relevant to asthma susceptibility in self‐reported AA individuals.

This study presents important novel detailed insights into differences associated with self‐reported racial background in asthma. These findings may help inform future strategies for targeted interventions and/or risk stratification in clinical practice. Importantly, our study also underscores the need for broader inclusions of diverse self‐reported racial groups in asthma research to promote equitable and biologically informed precision medicine healthcare solutions.

## 2 Results

### 2.1 Self‐Reported African American Asthma Patients Exhibit Elevated IL2‐STAT5 Signaling Across Multiple Compartments

To probe the potential relevance of self‐reported racial grouping in asthma, we exhaustively surveyed asthma‐related transcriptomic datasets available in Gene Expression Omnibus (GEO). We found that most studies did not account for ancestral backgrounds or self‐reported racial/ethnic groupings. Only eight studies included such information (Table 1), and even among these, reporting was limited to self‐reported racial/ethnic groups (referred to hereafter as *self‐reported racial groups* for simplicity), with no documentation of ancestral backgrounds. These observations highlighted the underrepresentation of ancestral/self‐reported racial factors in current asthma research, emphasizing an important knowledge gap in the literature.

**TABLE 1:** Overview of the asthma transcriptomic datasets used in current study.

Cross‐sectional studies					
GEO ID	Gender	Ancestral background	Age	Organ compartment	Refs.
GSE201955	59F 20M	47 African American 28 European American	39.65 ± 1.39	Bronchial epithelial cell	[23]
GSE85567	49F 22M	43 African American 28 European American	38.66 ± 1.50	Airway epithelial cell	[24]
GSE86430	6F 10M	7 African American 9 Hispanic	9 ± 0.40	CD4^+^ T cell	[25]
GSE69683	147F 76M	8 Black/African American 215 White/Caucasian	N/A	Blood	[26]
**Longitudinal study**					
GSE19301	N/A	10 African American/Black 104 European American/White	N/A	PBMC	[31]
**Studies excluded**					
GSE65204	Unclear self‐reported racial group classification				
GSE109455	Unclear self‐reported racial group classification				
GSE172368	Only one African American sample				

Among the eight identified studies, three were excluded due to low data quality, leaving five datasets (four cross‐sectional, one longitudinal studies) for further analyses (Table 1, Materials and Methods section). Notably, in most of these studies, individuals of self‐reported EA background comprised the majority of participants, with self‐reported AA individuals representing the second largest self‐reported racial group. Other self‐reported racial groups were significantly underrepresented and lacked sufficient sample size to perform meaningful statistical comparisons. Consequently, given the available data, our analysis primarily focused on the comparisons between self‐reported EA and AA individuals.

**FIGURE 1:**
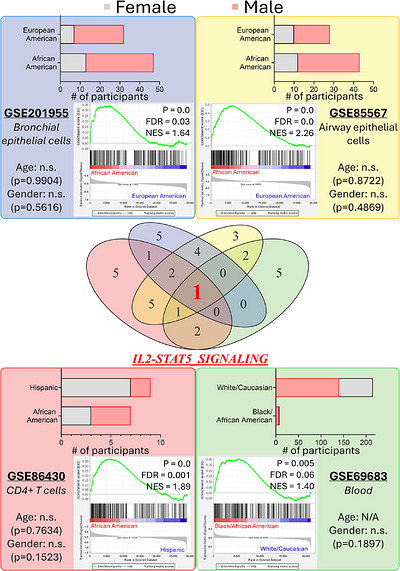
Gene set enrichment analysis (GSEA) of transcriptomic datasets of bronchial (blue), airway (yellow) epithelial cells, circulating CD4^+^ T cells (red), and whole blood (green), comparing individuals with asthma from self‐reported African American (AA) and other racial groups. Bar charts on the top showing the numbers of female (gray) and male (red) participants in each study. *p* values for comparing age profiles (*t*‐test) and gender compositions (chi‐square tests) of the studies were presented. Within GSEA plots, AA were shown as red on the left and other self‐reported racial groups were blue on the right. Venn diagram depicted that the IL2‐STAT5 signaling was the only pathway consistently enriched in AA samples relative to other self‐reported racial groups across all studies (FDR: false discovery rate; N/A: not available; NES: normalized enrichment score; n.s.: not significant; P: *p* value).

The four cross‐sectional studies included four different biological compartments: bronchial epithelium (47 self‐reported AA, 28 self‐reported EA, GSE201955) [23], airway epithelium (43 self‐reported AA, 28 self‐reported EA, GSE85567) [24], circulating CD4^+^ T cell (7 self‐reported AA, 9 self‐reported Hispanic, GSE86430) [25] and whole blood (8 self‐reported Black/AA, 215 self‐reported White/Caucasian/EA, GSE69683) [26]. In all studies, participants had physician‐confirmed asthma diagnoses prior to sample collection. Age and sex profiles did not differ significantly between self‐reported AA and other racial groups (Figure 1). Gene set enrichment analysis (GSEA) was performed on each dataset using the Hallmark Gene Set from GSEA [27]. Strikingly, across all four asthma datasets, IL2‐STAT5 signaling was significantly upregulated in self‐reported AA groups relative to other groups (Figure 1), highlighting a consistent pattern across multiple compartments. No consistent alteration was found for other signaling pathways across these datasets.

**FIGURE 2:**
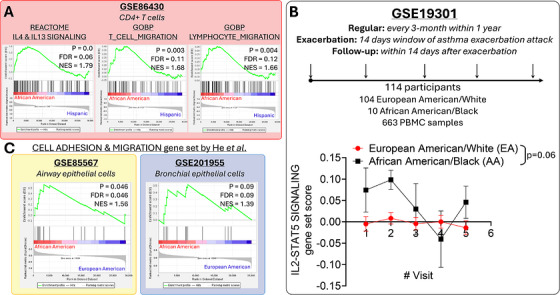
(A) GSEA for GSE86430 comparing circulating CD4^+^ T cells from individuals with asthma from African American (AA; red) versus Hispanic (blue) self‐reported racial groups, for IL4 and IL13 signaling (left), T cell migration (middle), and lymphocyte migration (right) gene sets. (B) Longitudinal analysis for GSE19301 for IL2‐STAT5 signaling gene set scores in peripheral blood mononuclear cells (PBMCs) from individuals with asthma from AA (Black) versus European American (EA, red) self‐reported racial groups by two‐way ANOVA. (C) GSEA for GSE85567 airway epithelial cell (left) and GSE201955 bronchial epithelial cell (right) datasets comparing AA (red) versus EA (blue) samples, for cell adhesion and migration signaling gene set reported by He et al. (FDR: false discovery rate; NES: normalized enrichment score; n.s.: not significant; P: *p* value).

Comparative analyses between asthma patients and healthy individuals further confirmed that IL2‐STAT5 pathway is significantly enhanced in asthma (Figure S1), consistent with previous reports highlighting its central role for asthma pathophysiology [28, 29, 30]. Notably, downstream of the IL2‐STAT5 axis, particularly, T helper 2 (Th2) cell responses like IL4 and IL13 signaling are also known to be critical in asthma. In line with this, the IL4 and IL13 signal gene set was enriched in self‐reported AA airway epithelial cells and circulating CD4^+^ T cells (Figure 2A and Table S1).

We next analyzed a longitudinal dataset comprising 104 self‐reported EA and 10 self‐reported AA asthma patients, followed over a 12‐month period (GSE19301) [31]. The relatively small number of AA participants compared with EA participants further underscore the persistent underrepresentation of these populations in asthma research. During this time, patients attended visits every 3 months, as well as additional visits during acute asthma exacerbation and a follow‐up 2 weeks later (Figure 2B). For consistency, our analyses focused on samples collected during the routine trimonthly visits. Gene set variation analysis (GSVA) [32] was performed on a total of 663 peripheral blood mononuclear cell (PBMC) samples to calculate their IL2‐STAT5 gene set scores over time. Longitudinally, self‐reported AA patients generally upregulated IL2‐STAT5 signaling relative to self‐reported EA patients (Figure 2B), trending toward statistical significance (*p* = 0.06). These findings support the cross‐sectional observations across other compartments and indicate that changes in IL2‐STAT5 signaling in self‐reported AA individuals is sustained over the course of asthma.

Mechanistically, an important role of the IL2‐STAT5 axis in asthma pathogenesis is the promotion of T cell migration [28]. In addition to the observed upregulating Th2‐associated signaling (Figure 2A and Table S1), increased IL2‐STAT5 signaling in CD4^+^ T cells from self‐reported AA patients was also associated with enrichment in gene sets linked to T cell and lymphocyte migration (Figure 2A). Likewise, airway and bronchial epithelium samples from self‐reported AA individuals exhibited elevated expression of gene sets reported by He et al. [28] related to cell adhesion and migration signals (Figure 2C). Together, these findings pinpointed that the heightened IL2‐STAT5 signaling in self‐reported AA patients may contribute to asthma pathophysiology by enhancing immune cell recruitment and trafficking to affected tissues.

Together, our multicompartment analyses revealed that, in asthma, self‐reported AA individuals consistently exhibit increased IL2‐STAT5 signaling. Upregulation of this pathway may contribute to enhanced T cell activation, and T cell migration and recruitment to the airways and bronchi. These findings might provide some insights related to the increased risk of asthma exacerbations observed among individuals from the self‐reported AA racial group [15, 16, 17, 18, 19, 20, 21].

### 2.2 Self‐Reported African American Healthy Individuals Exhibit Elevated IL2‐STAT5 Signaling in PBMCs

**FIGURE 3:**
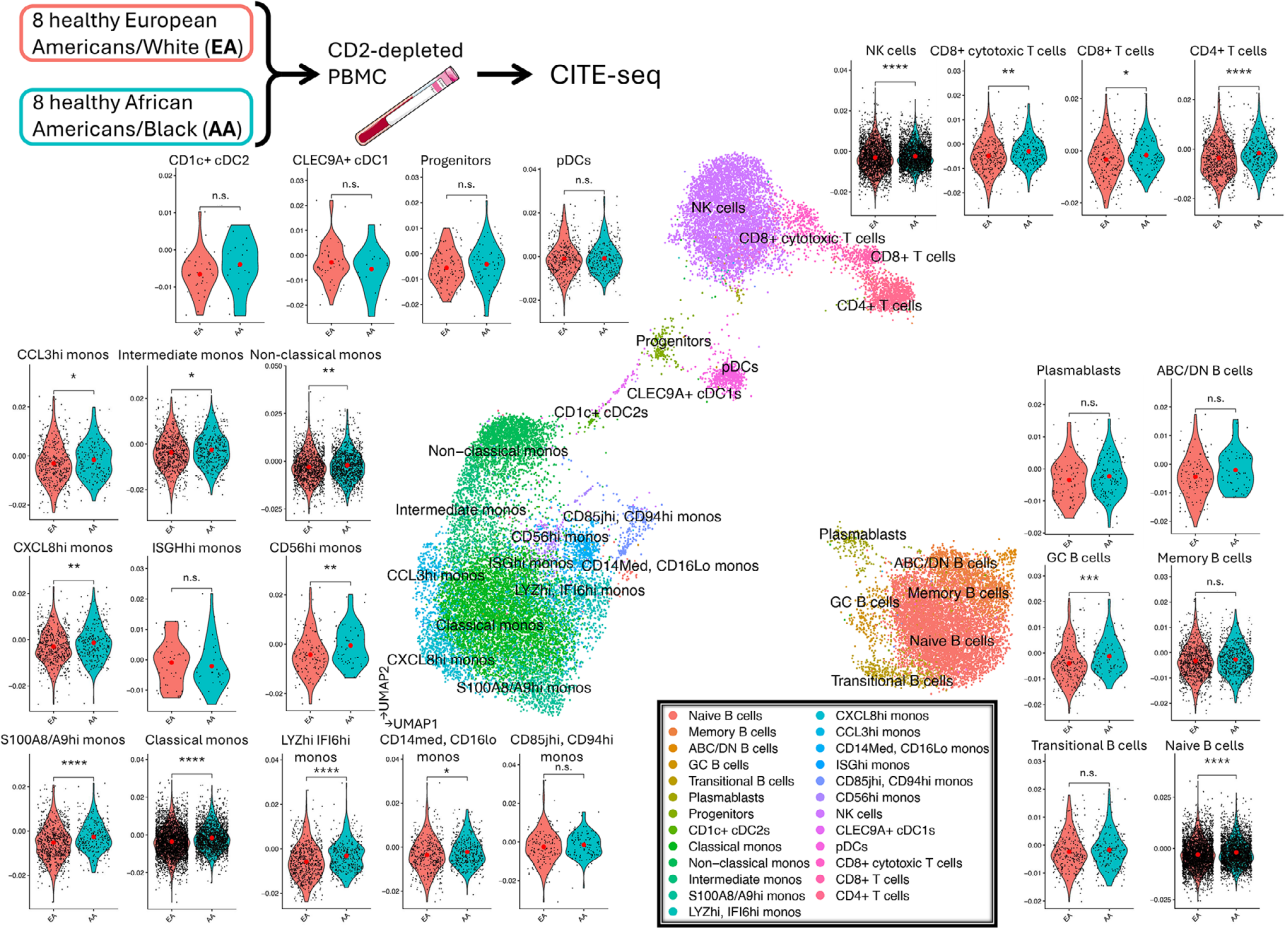
Cellular indexing of transcriptomes and epitopes sequencing (CITE‐seq) dataset analyzing PBMCs from healthy individuals with self‐reported African American/Black (AA) and European American/White (EA) backgrounds (GSE189050) projected onto the uniform manifold approximation and projection (UMAP) plot. Gene set scores for IL2‐STAT5 signaling were calculated for AA (cyan) and EA (pink), compared using Wilcoxon test, and shown as violin plots, with the red dots representing the average gene set score levels of the corresponding subsets. Parts of the image were provided by Servier Medical ART (https://smart.servier.com/), licensed under CC BY 4.0 (https://creativecommons.org/licenses/by/4.0/; ABC/DN B cells: age‐associated/double negative B cells; cDC: conventional dendritic cell; GC: germinal center; mono: monocytes; NK cells: natural killer cells; n.s.: not significant; pDC: plasmacytoid dendritic cell; *p < 0.05, **p < 0.01, ***p < 0.001, ****p < 0.0001).

We next investigated whether higher IL2‐STAT5 signaling is also found in nonasthmatic otherwise healthy self‐reported AA individuals. To address this, we analyzed a previously published cellular indexing of transcriptomes and epitopes sequencing (CITE‐seq) dataset (GSE189050) [12], comprising self‐reported healthy AA/Black (AA) and EA/White (EA) participants matched for age, sex, and body mass index (BMI; Figure 3). In this dataset, participants reported their self‐reported racial/ancestral backgrounds, which were further validated with genetic ancestry informative markers [33]. We compared the IL2‐STAT5 signaling across various PBMC subsets by calculating their gene set scores. A total of 25 immune cell subsets were identified in this CITE‐seq dataset and visualized using a uniform manifold approximation and projection (UMAP) plot (Figure 3). Strikingly, IL2‐STAT5 signaling was significantly elevated in self‐reported AA individuals across T and natural killer (NK) cell subsets, as well as in naive and germinal center (GC) B cells, and most monocyte populations (Figure 3). These findings suggest that, even under basal conditions, PBMC from self‐reported AA individuals generally exhibit upregulation of IL2‐STAT5 signaling pathway compared with those from self‐reported EA individuals.

**FIGURE 4:**
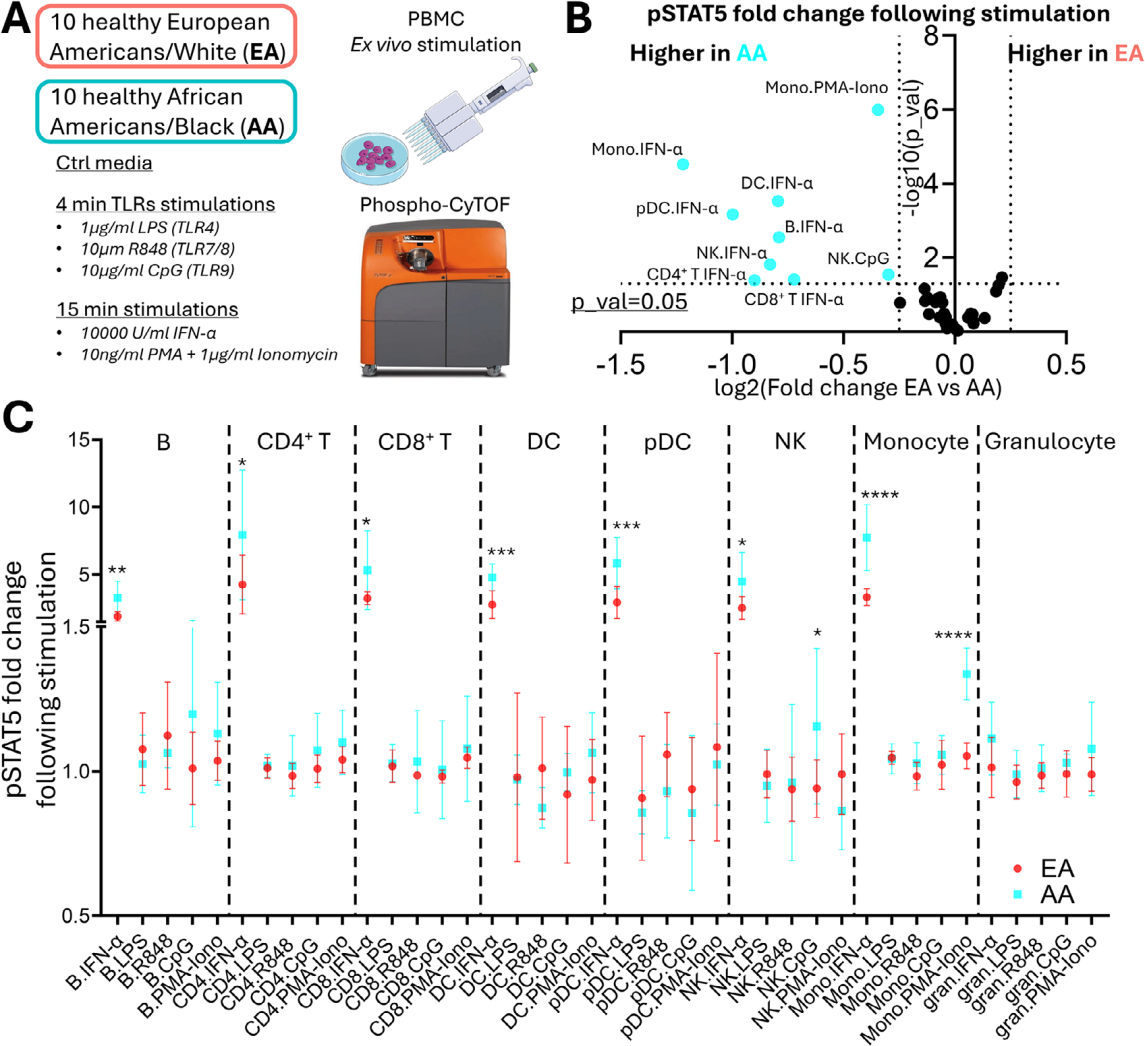
(A) Analysis of phospho‐cytometry by time‐of‐flight (CyTOF) data from Slight‐Webb et al. measuring activation profiles of PBMCs from healthy individuals of self‐reported African American/Black (AA) and European American/White (EA) backgrounds. Healthy AA and EA PBMCs were ex vivo stimulated under indicated conditions before measuring their STAT5 phosphorylation level (pSTAT5) by phospho‐CyTOF. Results were calculated as pSTAT5 fold changes after versus before stimulations. (B) Volcano plot visualizing the subsets that had statistically higher pSTAT5 fold changes in AA individuals (cyan) than EA individuals upon stimulation, with detailed comparisons presented in the dot plot below (C). Parts of the image were provided by Servier Medical ART (https://smart.servier.com/), licensed under CC BY 4.0 (https://creativecommons.org/licenses/by/4.0/; *p < 0.05, **p < 0.01, ***p < 0.001, ****p < 0.0001).

We next analyzed the phospho‐cytometry by time of flight (CyTOF) data from the same study [12]. Here, PBMC from AA and EA individuals were subjected to different stimuli, and STAT5 phosphorylation (pSTAT5) was quantified as an activation signal readout using CyTOF (Figure 4A). This approach enables assessment of activation thresholds across different immune cell subsets at a single cell level in response to stimulation. Across most PBMC subsets, samples from AA individuals showed significantly higher fold changes of pSTAT5 following stimulation compared with EA samples (Figure 4B). Such differences are particularly evident for IFN‐α stimulation (Figure 4C), which shares similar mechanisms for STAT5 activation to IL2 [34]. These results imply that IL2‐STAT5 signaling in AA‐derived PBMCs might be more readily activated, potentially underlying the enhanced IL2‐STAT5 pathway activity.

### 2.3 Self‐Reported African American Healthy Individuals Exhibit Elevated IL2‐STAT5 Signaling in Lung Immune Cells

**FIGURE 5:**
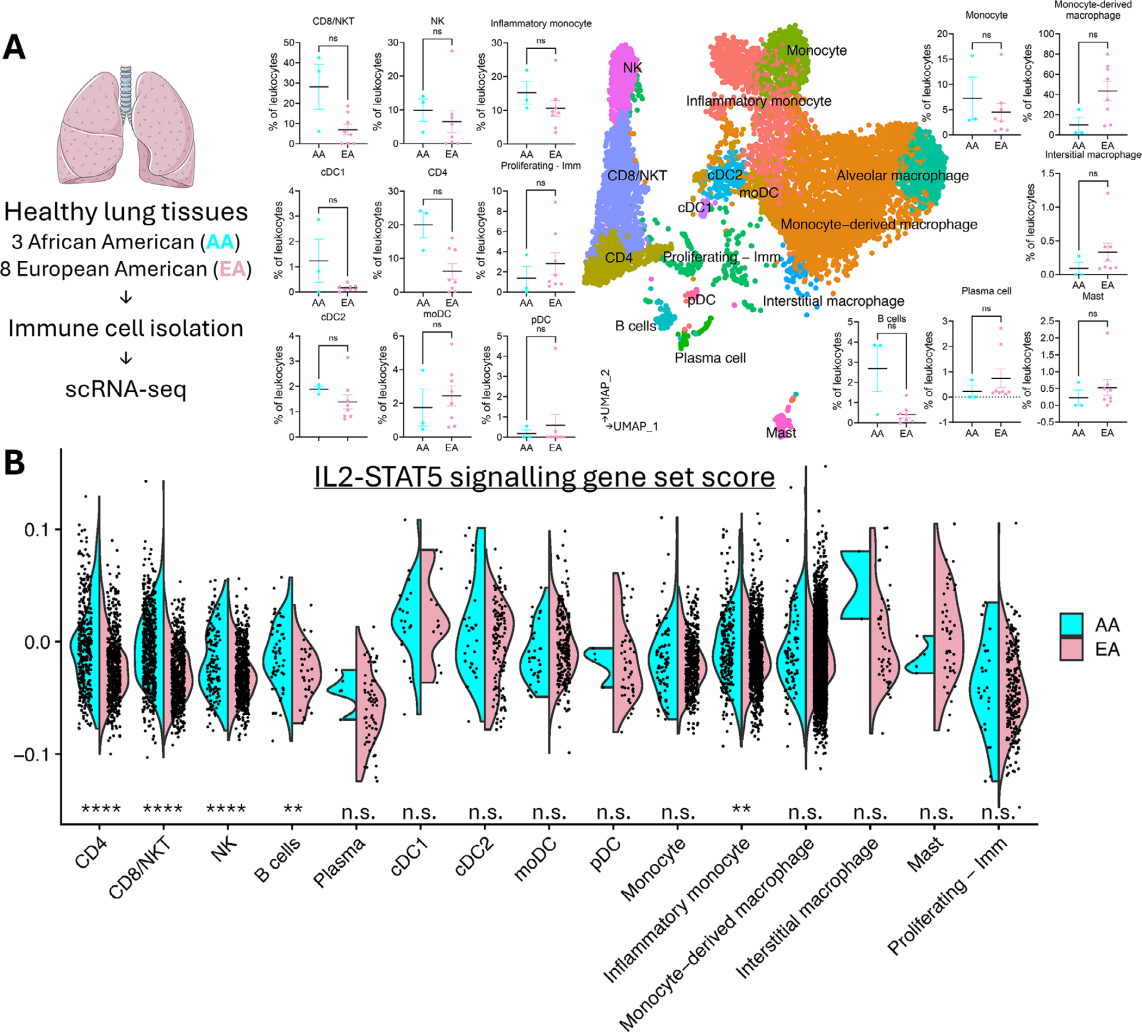
(A) Single cell RNA‐seq (scRNA‐seq) dataset analyzing lung‐derived immune cells from healthy individuals of self‐reported African American/Black (AA) and European American/White (EA) backgrounds (GSE227136) projected onto the uniform manifold approximation and projection (UMAP) plot. Cellular compositions were compared using Mann–Whitney test. (B) Gene set scores for IL2‐STAT5 signaling of the corresponding immune cell subsets were calculated for AA (cyan) and EA (pink), compared using Wilcoxon test, and shown as violin plots. Parts of the image were provided by Servier Medical ART (https://smart.servier.com/), licensed under CC BY 4.0 (https://creativecommons.org/licenses/by/4.0/; cDC: conventional dendritic cell; imm: immune cell; moDC: monocyte‐derived dendritic cell; NK: natural killer; n.s.: not significant; pDC: plasmacytoid dendritic cell; *p < 0.05, **p < 0.01, ***p < 0.001, ****p < 0.0001).

As the lungs are the primary organs affected by asthma, we next investigated whether the differences found in PBMCs were also present in lung‐resident immune cells. For this purpose, we analyzed a scRNA‐seq dataset of lung‐derived immune cells from healthy individuals of self‐reported AA and EA racial groups (GSE227136) [35]. A total of 16 immune cell subsets were captured in this dataset (Figure 5A). Alveolar macrophages were not detected in any self‐reported AA samples, likely reflecting technical artifacts and/or because fewer cells were sequenced (self‐reported AA: 1625 cells, self‐reported EA: 9647 cells). All other immune cell subsets were present in comparable proportions between self‐reported AA and EA individuals (Figure 5A). Gene set scores for IL2‐STAT5 signaling were computed across all immune cell subsets, excluding alveolar macrophages. Increased IL2‐STAT5 signaling in self‐reported AA samples was found in most adaptive immune cells, including T cells and B cells, as well as in some innate immune subsets such as NK cells, NKT cells and inflammatory monocytes (Figure 5B). Overall, these findings suggest that elevated IL2‐STAT5 signaling is also present in lung‐derived immune cells from healthy self‐reported AA compared to EA individuals, extending our observations made in peripheral blood.

## 3 Discussion

We present a comprehensive analysis examining detailed differences in asthma across different self‐reported racial groups. Our comprehensive analyses under both healthy and asthmatic conditions found that self‐reported AA individuals consistently demonstrated an upregulation of IL2‐STAT5 signaling across multiple organ compartments. Consistent with previous reports, such upregulation in IL2‐STAT5 signaling pathway was found to be associated with mediating activation and recruitment of T cells [28, 29, 30, 36, 37, 38, 39, 40], and airway and bronchial damage [28, 29, 30, 39, 40], and thus plausibly plays a role in more severe asthma exacerbation. Hence, heightened activity of this pathway may reflect a predisposition toward inflammatory responses during asthma for self‐reported AA individuals. Of note, while the observed differences in IL2‐STAT5 signaling were consistent, our analyses are of associative nature and do not necessarily establish causality.

Considering the critical involvements of IL2‐STAT5 signaling in T cell immunology like Th2 differentiation and migration [29, 30, 40, 41], it would be of interest to investigate whether the observed differences extend to other atopic diseases. In this context, we recently discovered that in atopic dermatitis, self‐reported EA patients exhibited higher IL4 and IL13 signaling relative to self‐reported Asian patients [14]. It remains to be studied whether the IL2‐STAT5 signaling pathway also plays a role in that context. Of note, in present study for self‐reported AA patients, we found compartment specific differences in downstream Th2‐associated signaling, with upregulated IL4 and IL13 pathway enrichment observed in airway epithelium and circulating CD4^+^ T cells, but not in bronchial epithelial cells and whole blood. Such distinct patterns likely mirror the organ specific immune responses, where resident versus circulating immune cells may differentially shape pathway activation, reflecting tissue specific regulations. However, further, more in‐depth and systematic studies are needed to delineate such differences across different organ systems. In this context, Th2 responses are also subject to modulations from other signals like the STAT3‐GATA3 pathway [42, 43]. Their detailed implications require further in‐depth interrogation.

Our analyses have several limitations. Asthma pathogenesis and treatment processes are multifactorial and are known to be influenced by various environmental factors [44, 45]. Specifically, self‐reported racial groups are social but not biological constructs. They represent an imperfect proxy, partially capturing the combined effects of the genetic ancestry, the exposome and the societal factors, that collectively can influence immunity and consequently, health and diseases. For example, self‐reported AA individuals might be disproportionately exposed to disadvantaged socioeconomic environments, which could contribute to their increased asthma susceptibility and reduced access to advanced treatments for asthma and other allergic disease [46, 47, 48, 49, 50]. Data on these environmental and socioeconomic factors were not included in any of the studies and datasets we surveyed. Future validation studies controlling for these confounders are warranted.

Additionally, the modest sample sizes and imbalanced sample compositions among different self‐reported racial groups as observed in some datasets represent a further limitation. Achieving statistical significance despite a relatively small and/or imbalanced sample sizes reinforces the robustness of the observed differences in IL2‐STAT5 signaling. On the other hand, the relatively small and/or imbalanced sample sizes might potentially influence the statistical power of the analyses, thereby plausibly limiting our ability to detect differences in other signal pathways. Nevertheless, in spite of these challenges, we provided both cross‐sectional and longitudinal comparisons and integrated results from independent studies across different organ compartments and using different analytical methodologies. This is expected to improve the robustness and reliability of our findings. Furthermore, in this regard, it is worth noting that in these publicly available datasets, with the exceptions of self‐reported AA and EA groups, other self‐reported racial groups were almost universally underrepresented. This highlights the necessity to include individuals of more diverse self‐reported racial backgrounds in future studies.

Collectively, our work provides the first in‐depth multicompartment analyses of immunological differences associated with asthma across self‐reported racial groups, with consistent enhanced IL2‐STAT5 signals observed in self‐reported AA individuals. Our study implies that self‐reported racial background might be an important factor to consider in asthma clinical research and potentially clinical management. With further validation, in better controlled prospective studies with broader and more diverse cohorts, our findings could help to inform asthma risk stratification and the development of more biologically informed therapeutic approaches toward precision medicine. Importantly, our research also highlights the urgent need for greater representation of diverse populations across all aspects of asthma research. Expanding inclusion will be essential to promote health equity and reduce health disparities in asthma.

## 4 Materials and Methods

### 4.1 Data Curation

We exhaustively surveyed transcriptomic studies in GEO, searching for transcriptomic datasets with relevant information or metadata related to ethnicity/race/ancestry information. All ethnicity/race/ancestry‐related data were collected from original published studies.

For transcriptomic datasets, eight asthma‐related studies with race/ethnicity/ancestry‐related information were found on GEO as in Table 1. Two studies did not report clear racial/ancestral classification (GSE65204, GSE109455) and one study only contained one self‐reported AA sample (GSE172368). They are thus excluded. The remaining five datasets of high data quality and good sample racial/ancestral background coverage were subject to analyses using their corresponding normalized data (GSE201955, GSE85567, GSE86430, GSE69683, GSE19301). For the longitudinal dataset (GSE19301), patients attended regular visits every 3 months and during acute asthma exacerbation and a 2‐week follow‐up visit after the exacerbation. We only focused on the regular visit timepoints for their better data coverage and more consistent comparison, as samples from exacerbation and follow‐up timepoints were sporadic and analysis might be affected by bias or outliers.

The CITE‐seq and single cell RNA‐sequencing (scRNA‐seq) datasets analyzing PBMCs and lung‐derived immune cells from healthy individuals of self‐reported AA/Black (AA) and EA/White (EA) racial groups was downloaded from the respective original publication [12, 35] (GEO ID: GSE189050, GSE227136).

For phospho‐CyTOF data, the 25%, 50% (median), and 75% quantile measurements were available from the original paper [12]. Their corresponding means and standard deviations were estimated based on a method previously described [51], with means calculated as the average value of 25%, 50% (median), and 75% quantile measurements and standard deviations calculated as the differences between 75% and 25% quantile measurements divided by 1.35.

### 4.2 Ethics Statement

For GSE201955 [23], GSE85567 [24], the studies were approved by the University of Chicago's Institutional Review Board, and written informed consent was obtained from all participants. For GSE86430 [25], the study was approved by the institutional review board at Albert Einstein College of Medicine. GSE69683 [26] was registered as a multicenter prospective cohort study involving 16 clinical centers in 11 European countries (NCT01982162). It was approved by the Institutional Review Boards of all the participating institutions and written informant consents were provided by all participants. GSE19301 [31] was a multicenter prospective noninterventional study carried out in Australia, Ireland, Iceland, United Kingdom, and United States. It was approved by the Institutional Review Boards or Ethics Committees of all the institutions involved. Written informed consents were obtained from all participants.

Study generating CITE‐seq data (GSE189050) and phospho‐CyTOF data [12] was approved by the Institutional Review Board at Oklahoma Medical Research Foundation. Written informed consents were provided by all participants.

For GSE227136 [35], the study was approved by the local Institutional Review Boards (Vanderbilt IRB nos. 060165 and 171657; Western IRB no. 20181836). Written informed consent was obtained from all participants.

The authors of this study have consulted with the Chair of the Human Research Ethics Committee of the Nepean Blue Mountains Local Health District Network, who advised that no ethical review is required from the committee for this study.

### 4.3 Self‐Reported Racial/Ethnic/Ancestral Profiling

Self‐reported racial/ethnic/ancestral background information of the datasets that we analyzed was all retrieved from the original publications. Most studies documented the self‐reported race or ethnicity of the participants, which we referred to as *self‐reported racial group* for simplicity. Notably, for CITE‐seq dataset (GSE189050) and phospho‐CyTOF dataset from work by Slight‐Webb et al., participants reported their self‐reported racial/ancestral backgrounds, which was further validated using genetic ancestry informative markers [33].

### 4.4 Bioinformatic and Statistical Analysis

For RNA‐seq datasets, age profiles of each self‐reported racial group (e.g., AA vs. EA) were compared using *t*‐test and gender profiles of them were compared with chi‐square tests using GraphPad PRISM (*v11.0.0*). In all datasets, these profiles among each self‐reported racial group were all comparably balanced, with no significant difference.

Transcriptomic datasets were analyzed with GSEA software (*v4.3.2*) [27] following its tutorial, which computationally determines whether a priori defined sets of genes are statistical different between two conditions (e.g., two self‐reported racial groups, AA vs. EA), with false discovery rate (FDR) adjustments. Here, analyses were based on the Hallmark Gene Set curated by GSEA (https://www.gsea‐msigdb.org/gsea/msigdb/human/genesets.jsp?collection=H). For the analysis for “CELL_ADHESION & MIGRATION” gene set, the corresponding genes were adapted from the original publication by He et al. [28]. For all GSEA, FDR < 0.25 was considered to be statistically significant. In the longitudinal study analysis, GSVA (*v1.51.5*) [32] was run following its tutorial, based on the “IL2_STAT5_SIGNALING” gene set from the GSEA Hallmark Gene Set.

CITE‐seq and scRNA‐seq data were analyzed with *Seurat v4.3.0* [52]. In brief, cell subsets were annotated based on the original publications and gene set scores were calculated using the *CellCycleScoring* function in *Seurat* following its tutorial based on the “IL2_STAT5_SIGNALING” gene set documented in GSEA (https://www.gsea‐msigdb.org/gsea/msigdb/human/search.jsp) [27]. Cellular compositions were compared using Mann–Whitney test. Gene set scores of immune cell subsets from different groups were compared with Wilcoxon test.

For phospho‐CyTOF data, AA versus EA comparisons were performed with GraphPad PRISM (*v11.0.0*) and the ones with *p* values lower than 0.05 and log2(fold change EA vs. AA) bigger than 0.25 or smaller than −0.25 were considered to be statistically significant.

## Author Contributions

**Duan Ni and Ralph Nanan**: concept and design; **Duan Ni and Ralph Nanan**: acquisition, analysis and interpretation of data; **Duan Ni and Ralph Nanan**: drafting of the manuscript; **Duan Ni and Ralph Nanan**: critical revision of the manuscript for important intellectual content. Both the authors have read and approved the final manuscript.

## Funding

This project is supported by the Norman Ernest Bequest Fund.

## Ethics Statement

Ethics statements for the original studies were described in Materials and Methods section.

The authors have consulted this study with the Chair of the Human Research Ethics Committee of the Nepean Blue Mountains Local Health District Network, who advised that no ethical review is required from the committee for this study.

## Conflicts of Interest

Ralph Nanan has served on a scientific advisory board of Sanofi. Duan Ni has no conflicts of interest to declare.

## Supporting information




**Supporting Information**: mco270810‐sup‐0001‐SuppMat.pdf

## Data Availability

All data used in this study are publicly available from the original publications as described in Materials and Methods section. They are openly available in Gene Expression Omnibus (GEO, https://www.ncbi.nlm.nih.gov/geo/) with IDs: GSE201955, GSE85567, GSE86430, GSE69683, GSE19301, GSE189050, GSE227136. Phospho‐CyTOF data were obtained from the original publication Supporting Information Data.
