# Supplementary material for: Cross‐Compartment Multimodal Analyses Reveal Differences in IL2‐STAT5 Signaling Associated With Asthma in Individuals From Diverse Backgrounds
Source: MedComm (2020). 2026 Jun 11;7(6):e70810. doi: 10.1002/mco2.70810 (PMC13260699; doi:10.1002/mco2.70810)
Supplement: Supplementary file 1 — Supporting Information: mco270810‐sup‐0001‐SuppMat.pdf [file MCO2-7-e70810-s001.pdf]

Cross-compartment multi-modal analyses reveal differences in IL2-STAT5 signalling associated with asthma in individuals from diverse backgrounds

Duan Ni[1,2,3], Ralph Nanan[1,2,3*]

1 Sydney Medical School Nepean, The University of Sydney, Sydney, NSW, Australia

2 Charles Perkins Centre, The University of Sydney, Sydney, NSW, Australia

3 Nepean Hospital, Nepean Blue Mountains Local Health District, Sydney, NSW, Australia

*Correspondence:

Ralph Nanan

ralph.nanan@sydney.edu.au

Sydney Medical School Nepean, The University of Sydney.

Nepean Hospital, Level 5, South Block, Penrith NSW, 2751, Australia

Telephone: +61 2 4734 1614

Fax: +61 2 4734 1144

Supplementary Information

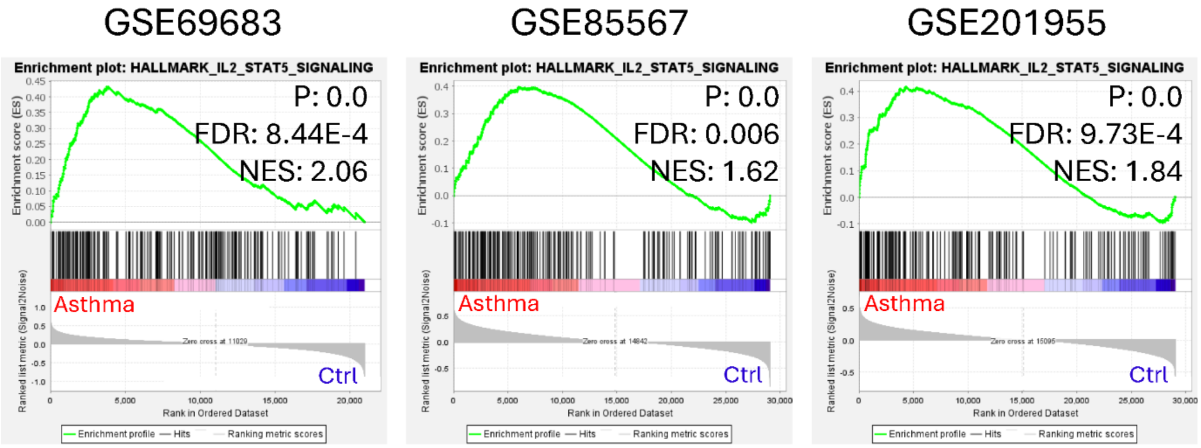

Figure S1. Gene set enrichment analysis (GSEA) plots for "IL2_STAT5_SIGNALING" gene set in GSE69683 (whole blood), GSE85567 (airway epithelial cells) and GSE201955 (bronchial epithelial cells) comparing asthma and health control samples.

Table S1. Gene set enrichment analysis results for "REACTOME_INTERLEUKIN_4_AND_INTERLEUKIN_13_SIGNALING" gene set across 4 asthma transcriptomic datasets.

GEO ID	Organ compartment	Normalized enrichment score	P value	FDR q value
GSE201955	Bronchial epithelial cell	n.s.	n.s.	n.s.
GSE85567	Airway epithelial cell	1.72	0.0	0.04
GSE86430	$CD4^+$ T cell	1.79	0.0	0.06
GSE69683	Whole blood	n.s.	n.s.	n.s.
